# Knowledge, attitudes, and practices related to the COVID-19 pandemic among pregnant women in Bangkok, Thailand

**DOI:** 10.1186/s12884-022-04612-3

**Published:** 2022-04-23

**Authors:** Jadsada Kunno, Pataraporn Yubonpunt, Busaba Supawattanabodee, Chavanant Sumanasrethakul, Budsaba Wiriyasirivaj

**Affiliations:** 1 Department of Research and Medical Innovation, Faculty of Medicine Vajira Hospital, Navamindradhiraj University, Bangkok, Thailand; 2 Department of Public Health, Faculty of Public and Environmental Health, Huachiew Chalermprakiet University, Samut Prakan, Thailand; 3 Department of Urban Medicine, Faculty of Medicine Vajira Hospital, Navamindradhiraj University, Bangkok, Thailand; 4 Department of Obstetrics and Gynecology, Faculty of Medicine Vajira Hospital, Navamindradhiraj University, Bangkok, Thailand

**Keywords:** Knowledge, Attitudes, Practices, COVID-19 pandemic, Pregnant, Thailand

## Abstract

**Background:**

Pregnancy is associated with increased risk for severe COVID-19. Few studies have examined knowledge, attitudes, and practices (KAP) related to pregnancy during the pandemic. This study investigated the association between socio-demographic characteristics and KAP related to COVID-19 among pregnant women in an urban community in Thailand.

**Methods:**

A cross-sectional online survey was distributed among pregnant women in Bangkok, Thailand from July–August 2021. Binary logistic regression was conducted to test the association between socio-demographic characteristics and KAP related to COVID-19, and a Spearman’s analysis tested correlations between KAP scores.

**Results:**

A total of 150 pregnancy survey responses were received. Most participants were third trimester (27–40 weeks gestation; 68.0%). Pregnancy had never been risked contracting COVID-19 (84.7%). Most expressed concerns about being infected with COVID-19 during pregnancy and following birth (94.0 and 70.0%, respectively). The results of binary logistic regression analysis found associations between knowledge and marital status (OR = 4.983, 95%CI 1.894–13.107). In addition, having a bachelor’s degree or higher was associated with higher attitude scores (OR = 2.733, 95%CI 1.045–7.149), as was being aged 26–30 (OR = 2.413 95%CI 0.882–6.602) and 31–35 years of age (OR = 2.518–2.664, 95%CI 0.841–8.442). Higher practice scores were associated with having a bachelor’s degree or higher (OR = 2.285 95%CI 1.110–6.146), and income ≥15,001 bath (OR = 4.747 95%CI 1.588–14.192). Correlation analysis found a weak positive correlation between knowledge and practice scores (*r* = 0.210, *p*-value = 0.01).

**Conclusion:**

Participants overall had high KAP scores. This study can guide public health strategies regarding pregnant women and COVID-19. We recommend that interventions to improve and attitude and practice scores. Knowledge on pregnancy and COVID-19 should focus on reducing fear and improving attitudes toward the care of patients as well as the promotion of preventive practices.

**Supplementary Information:**

The online version contains supplementary material available at 10.1186/s12884-022-04612-3.

## Introduction

Coronavirus 2019 (COVID-19) has been deemed a global health emergency [1]. As of August 2021, Thailand has experienced the highest numbers of new cases in South and Southeast Asia, with a 26% increase compared with 7% in India and 5% in Indonesia [2]. Most people infected with the COVID-19 virus will experience mild-to-moderate respiratory illness and recover without requiring special treatment [3].

Pregnant women may be at increased risk for severe COVID-19 illness [4–6]. To reduce the chances of infection, pregnant women should be aware of their potential risk and preventative measures [5], and potential barriers to their adherence to such measures should be addressed [7]. Although pregnant women infected with COVID-19 have been found to be less likely to have symptoms compared with non-pregnant women in similar age groups, studies have indicated that they are at increased risk for ICU admission and more likely to experience preterm labor, and their newborns are more likely to be in a neonatal ICU [4]. Moreover, cases have been reported of COVID-19-related complications such as hydrops fetalis and intrauterine fetal demise [8], which are possibly related to changes in infants’ immune system and respiratory physiology [9]. Although limited data are available about COVID-19 during pregnancy, information on the impacts of other highly pathogenic coronaviruses such as severe acute respiratory syndrome and the Middle East respiratory syndrome on pregnancy can provide insights into COVID-19 effects [10]. Such viruses may predispose pregnant women to higher risks of severe disease and poorer neonatal outcomes [11]. Thus, a better understanding of knowledge, attitudes and practices around COVID-19 among pregnant women and mothers of infants is needed [12].

People’s adherence to infection control measures will be largely affected by their knowledge, attitude, and practices (KAP). “KAP theory” is a health behavior change framework wherein factors contributing to human behavioral change are divided into three successive processes, namely the acquisition of proper knowledge, generation of attitudes, and adoption of behaviors (or practices) [13]. The public’s KAP play a major role in the prevention and control of infectious diseases [14]: thus, it is expected that KAP levels will be a deciding factor in the battle against COVID-19. The public must routinely practice precautionary behaviors to control the spread of COVID-19 and requiring people to adhere to social distancing and appropriate preventative practices can help prevent or contain outbreaks [11, 15]. However, effective pandemic management requires an adequate understanding of the factors that influence behavioral changes [16], including the ways that KAP affect individuals’ adherence to government measures [17]. As the same time knowledge about COVID-19 infection in pregnant women and newborns is scarce [18].

The issue of pregnancy during this global pandemic deserves a more sensitive approach and mutual understanding among clinicians and other healthcare workers; however, A few studies assessing attitudes and perceptions of the effect of COVID-19 among pregnant women [19–22]. As the COVID-19 pandemic continues to intensify globally, it is important to understand the mentality of pregnant women towards COVID-19. The only preventive measures available are social distancing, hand washing and face masks; however, few studies have focused on KAP. To help address this gap, this paper reports the results of a cross-sectional survey of pregnant women in Bangkok, Thailand. Specifically, this study aimed to: 1) collect information on pregnant women’s baseline knowledge, attitudes, and practices related to COVID-19; 2) examine potential associations between socio-demographic characteristics and COVID-19-related knowledge, attitudes, and practices, and 3) investigate possible correlations between knowledge, attitude, and practice dimensions. In so doing, this study can help identify various characteristics of pregnant women who are more likely to be vulnerable to the effects of COVID-19.

## Methods

### Study design

This study entailed the analysis of a cross-sectional survey distributed among pregnant women attending the obstetrics and gynecology clinic at the Faculty of Medicine Vajira Hospital, Bangkok, Thailand from July–August 2021. The study was approved by the ethics committee of the Faculty of Medicine Vajira Hospital, Navamindradhiraj University, Bangkok, Thailand. (COA 116/2564). All experiments were performed in accordance with relevant guidelines and regulations, in the Declarations section.

### Participants

Pregnant women aged 18 years and older living in Bangkok, Thailand availing themselves of gynecology and obstetrics services at Faculty of Medicine Vajira Hospital, Bangkok were eligible to participate in the study. The sample size was calculated using G*Power based on the estimated population of pregnant women in the city.

### Data collection

Data was collected using an online survey distributed on social media using a snowball technique. The invitation asked participants to confirm that informed consent was obtained from all subjects, voluntary participation and provided instructions for filling in the questionnaire. All participants have been performed in accordance with the Declaration of Helsinki and have been approved by an appropriate ethics committee.

### Questionnaire

The questionnaire takes about 10 min to complete and is divided into four sections. (see Supplementary files 1 for details). The questions were designed and modified by an expert team of researchers and previous study [11, 23]**.** The first section collected socio-demographic information, including age, occupation, education level, marital status, income, and religion, in addition to clinical characteristics such as pregnancy trimester, number of pregnancies, type of conception, history of miscarriages, current and previous pregnancy complications, settlement type, and questions designed to gauge participants’ risk of contracting COVID-19 (see Table 1 for details). The knowledge section consists of 13 items (K1–K13) scored as False = 0 and True = 1. Knowledge item responses were summed to a total score from 0 to 13 and the cut-off point for adequate/inadequate overall knowledge was set at 60% (≥ 9 points). The Attitudes section consisted of 17 items (A1–A17) assessing perceptions of whether the country could win the fight against the COVID-19 pandemic, for which responses were scored as Agree = 2, Not Sure = 1, and Disagree = 0. Attitude item responses were summed to a total score ranging from 0 to 34, and the cut-off point for overall favorable/unfavorable attitudes was set at 27 points. The Practices section consisted of 11 questions (P1–P11) scored as Practiced = 2, Not sure = 1, and Not practiced = 0. Practice item responses were summed to a total score ranging from 0 to 22, and the cut-off point for proper/improper practices was set at 18 points. Practice items were based on perceptions of whether the country could win the fight against the COVID-19 pandemic (see Table 2 for details).

**Table 1:** Characteristics of participants (*n* = 150)

Socio-demographic characteristics	n (%)
**Pregnancy**	150 (100.0%)
**Age (years)**	
18–25	46 (30.7%)
26–30	43 (28.7%)
31–35	32 (21.3%)
≥ 36	29 (19.3%)
**Occupation**	
Civil servant	18 (12.0%)
Employee	87 (58.0%)
Housewife	45 (30.0%)
**Education**	
< Bachelor’s degree	104 (69.3%)
≥ Bachelor’s degree	46 (30.7%)
**Status**	
Unmarried	41 (27.3%)
Married	109 (72.7%
**Income (Thai baht)**	
< 15,000	101 (67.3%)
≥15,000	49 (32.7%)
**Religion**	
Buddhism	146 (97.3%)
Christianity	1 (0.7%)
Islam	3 (2.0%)
**Trimester**	
First ≤12 weeks gestation	12 (8.0%)
Second 13–26 weeks gestation	36 (24.0%)
Third 27–40 weeks gestation	102 (68.0%)
**Number of pregnancies**	
1	64 (42.7%)
2	49 (32.7%)
3	27 (18.0%)
≥4	10 (6.7%)
**Type of conception**	
Naturally conceived	148 (98.7%)
Not naturally conceived	2 (1.3%)
**Number of miscarriages**	
0	124 (82.7%)
1	23 (15.3%)
≥2	3 (2.0%)
**Current complications**	
No	127 (84.7%)
Yes	23 (15.3%
**Previous complications**	
No	135 (90.0%)
Yes	15 (10.0%)
**Settlement type**	
Urban	150 (100.0%)
**Risk of contracting COVID-19**	
Infection COVID-19	1 (0.7%)
At risk (ever had a screening test)	8 (5.3%)
Never at risk	127 (84.7%)
Not sure	14 (9.3%)

**Table 2:** Frequency scores for knowledge, attitudes, and practice items

**Knowledge items**		**n (%)**		
		**True**		**False**
K1	Human-to-Human transmission of COVID-19	142 (94.7%)		8 (5.3%)
K2	COVID-19 can be spread by droplets and aerosols.	134 (89.3%)		16 (10.7%)
K3	COVID-19 symptoms include mild fever, tiredness, dry cough, and muscle pain.	135 (90.0%)		15 (10%)
K4	Everyone has the same risk of infection from COVID-19.	126 (84.0%)		24 (16.0%)
K5	There currently is no treatment for COVID-19.	97 (64.7%)		53 (35.3%)
K6	The pneumococcal vaccine can protect against COVID-19.	25 (16.7%)		125 (83.3%)
K7	Not everyone with COVID-19 will have severe symptoms. But people with underlying or chronic diseases are more likely to have severe symptoms.	111 (74.0%)		39 (26.0%)
K8	Wearing two of masks can prevent infection from COVID-19 better than one layer.	96 (64.0%)		54 (36.0%)
K9	Wash your hands frequently	128 (85.3%)		22 (14.7%)
K10	Regular rinsing nasal mucus with saline can prevented with COVID-19.	38 (25.3%)		112 (74.7%)
K11	A person who comes into contact with someone infected with COVID-19 should be isolated immediately in 14 days.	137 (91.3%)		13 (8.7%)
K12	Vaccination against COVID-19 prevents severe symptoms.	88 (58.7%)		62 (41.3%)
K13	There currently is no information on the efficacy and safety of vaccinations against COVID-19.	69 (46.0%)		81 (54.0%)
**Knowledge mean ± SD = 9 ± 2.3; Max score = 13**				
**Attitudes items**		**n (%)**		
		**Agree**	**Not Sure**	**Disagree**
A1	COVID-19 can be controlled	33 (22.0%)	99 (66.0%)	18 (12.0%)
A2	COVID-19 has affected daily life.	142 (94.7%)	6 (4.0%)	2 (1.3%)
A3	Pregnant women may have a higher chance than other populations of being infected with COVID-19.	133 (88.7%)	16 (10.7%)	1 (0.7%)
A4	Pregnancy may increase the risk of respiratory failure than other population.	105 (70.0%)	43 (28.7%)	2 (1.3%)
A5	I am concerned about being infected with COVID-19 during pregnancy.	141 (94.0%)	9 (6.0%)	0
A6	I am concerned about being infected with COVID-19 following pregnancy.	105 (70.0%)	39 (26.0%)	6 (4.0%)
A7	Do you think the fetus can be infected?	78 (52.0%)	66 (44.0%)	6 (4.0%)
A8	Do you think your baby can be infected after birth?	78 (52.0%)	69 (46.0%)	3 (2.0%)
A9	Do you think the baby can be infected during delivery?	80 (53.3%)	68 (45.3%)	2 (1.3%)
A10	Contracting COVID-19 during pregnancy may increase the risk of miscarriage	83 (55.3%)	65 (43.3%)	2 (1.3%)
A11	Do you agree if your doctor will be advised you for caesarean section over a vaginal delivery if you are diagnosed with COVID-19?	94 (62.7%)	56 (37.3%)	0
A12	If you are diagnosed have COVID-19, how likely do you think is the risk of infection to the baby after delivery	44 (29.3%)	58 (38.7%)	48 (32.0%)
A13	If infected with COVID-19 after delivery, will you isolate yourself for 2 weeks?	116 (77.3%)	30 (20%)	4 (2.7%)
A14	Will you breastfeed by yourself?	139 (92.7%)	7 (4.7%)	4 (2.7%)
A15	If infected with COVID-19 after delivery, will you breastfeed?	16 (10.7%)	57 (38.0%)	77 (51.3%)
A16	Do you want to be vaccinated against COVID-19 during pregnancy?	79 (52.7%)	51 (34.0%)	20 (13.3%)
A17	Do you want to be vaccinated against COVID-19 during breastfeeding?	81 (54.0%)	48 (32.0%)	21 (14.0%)
**Attitudes mean ± SD = 27 ± 3.9, Max score = 34**				
**Practice items**		**n (%)**		
		**Practiced**	**Not sure**	**Not practiced**
P1	Will/have been tested for COVID-19 during pregnancy	52 (34.7%)	9 (6.0%)	89 (59.3%)
P2	Will be tested if you experience COVID-19 symptoms during pregnancy	133 (88.7%)	15 (10.0%)	2 (1.3%)
P3	You wear a mask every time you leave the house.	149 (99.3%)	1 (0.7%)	0
P4	You frequently wash your hands or clean them with alcohol.	149 (99.3%)	1 (0.7%)	0
P5	You cover your mouth and nose with your elbow or a cloth or tissue when you cough or sneeze	135 (90.0%)	8 (5.3%)	7 (4.7%)
P6	You maintain at least 1 m distance from others in public places.	138 (92.0%)	11 (7.3%)	1 (0.7%)
P7	You avoid crowds and public places.	137 (91.3%)	9 (6.0%)	4 (2.7%)
P8	You follow news updates about COVID-19 situation	139 (92.7%)	5 (3.3%)	6 (4.0%)
P9	You have been to a risky place in the last 5 days	22 (14.7%)	24 (16.0%)	104 (69.3%)
P10	You have left the house wearing a mask within the last 5 days	131 (87.3%)	6 (4.0%)	13 (8.7%)
P11	You have strictly complied with government announcements.	142 (94.7%)	5 (3.3%)	3 (2.2%)
**Practice mean ± SD = 18 ± 1.9, Max score = 22**				

### Statistical analysis

In addition to descriptive statistics of the characteristics and dispersion measures (mean and standard deviation). Binary logistic regression was used to test the association between socio-demographic characteristics and COVID-19-related knowledge, attitudes, and practiced. Spearman’s correlation analysis was used to examine associations between knowledge, attitude, and practice scores. The level of statistical significance was set at *p*-value < 0.05 was considered to indicate statistical significance. The statistical analysis was performed using the Statistical Package for the Social Sciences Program (SPSS), version 22 (IBM Corp., Armonk, NY, USA).

## Results

A total of 150 questionnaire responses were obtained, and socio-demographic and clinical characteristics are presented in Table 1. The largest group of participants (30.7%) was aged 18–25 years, and the majority were married (72.7%), Buddhists (97.3%), and private employees or civil servants (70.0%), had attained less than a bachelor’s degree (69.3%), and reported incomes less than 15,000 Thai-bath (67.3%). These results indicate that population was representative of the wider population of pregnant women residing in Bangkok.

In term of clinical characteristics, the majority of participants were in their third trimester (27–40 weeks gestation; 68.0%), and over 40% were in their first pregnancy. Most pregnancies were naturally conceived (98.7%). The majority of participants reported no history of miscarriage (82.7%), had experienced no current or previous complications (84.7 and 90%, respectively). In addition, most reported that they had never been in a situation in which they risked contracting COVID-19 (84.7%).

### Knowledge, Attitude, and Practice scores on COVID-19

Participants’ knowledge, attitude, and practice item scores were showed in Table 2**.** The mean knowledge score was (9 ± 2.3). Nearly 90% of participants knew that COVID-19 can be spread by droplets and aerosols (K2), and 65% knew that there is currently no treatment for COVID-19 (K5). Over 83% responded that the pneumococcal vaccine cannot protect against COVID-19 (K6), and 75% reported that regular rinsing nasal mucus with saline cannot prevent infection (K10). However, 64% responded that wearing two masks are more effective than a single mask to prevent infection (K8).

The mean attitudes score was (27 ± 3.9). The majority (66.0%) of participants were not sure if COVID-19 could be controlled (A1), agreed that the pandemic had affected daily life 142 (94.7%) (A2), and agreed that pregnant women were at higher risk of infection a chance than other populations 133 (88.7%) (A3). Most expressed concerns about being infected with COVID-19 during pregnancy and following birth (94.0 and 70.0%, respectively) (A5-A6). In addition, 38.7% were not sure if diagnosed have COVID-19, is the risk of infection to the baby after delivery (A12), and 77.3% agreed that they would isolate themselves for 2 weeks if they became infected with COVID-19 (A13). Most participants agreed that they would breastfeed by themselves (92.7%) (A14) and indicated that they would not breastfeed if infected with COVID-19 after delivery (51.3%) (A15).

The mean practice score was (18 ± 1.9). The majority of participants (99.3%) stated that they wore a mask every time they left the house (P3) and frequently washed their hands or cleaned them with alcohol (P4). Most participants claimed to avoid crowds and public places (91.3%) (P7) and follow news updates about the COVID-19 situation (92.7%) (P8).

### Associations between knowledge and socio-demographic characteristics

Table 3 shows the bivariate analysis of knowledge scores and socio-demographic characteristics. The variables of being a housewife and married status were significantly associated with knowledge levels, as were all age groups (*p*-value < 0.05). In addition, having a bachelor’s degree or higher was likely associated with knowledge (*p*-value = 0.061). The results of the multivariate analysis indicated a significant association between marital status and knowledge about COVID-19 (*p*-value = 0.001), whereby married participants had greater knowledge than those who were unmarried (OR = 4.983, 95%CI 1.894–13.107).

**Table 3:** Bivariate and multivariate analysis of knowledge scores and socio-demographic characteristics

Knowledge				
**Factor variables**	**Bivariate**		**Multivariate**	
	**OR (95%CI)**	***p*-value**	**OR (95%CI)**	***p*-value**
**Occupation**				
Civil servant	Ref.		Ref.	
Employee	0.380 (0.102–1.417)	0.150	0.511 (0.120–2.179)	0.364
Housewife	0.191 (0.049–0.753)	**0.018**	0.443 (0.090–2.173)	.316
**Age (years)**				
18–25	Ref.		Ref.	
26–30	2.693 (1.135–6.390)	**0.025**	1.450 (0.532–3.953)	0.467
31–35	3.322 (1.264–8.731)	**0.015**	1.044 (0.315–3.461)	0.911
≥ 36	4.086 (1.457–11.457)	**0.007**	1.413 (0.419–4.769)	0.578
**Education (degree)**				
< Bachelor	Ref.		Ref.	
≥ Bachelor	2.078 (0.967–4.463)	0.061	1.462 (0.524–4.769)	0.468
**Status**				
Unmarried	Ref.		Ref.	
Married	6.231 (2.840–13.670)	**0.01**	4.983 (1.894–13.107)	**0.001**
**Income (Thai baht)**				
< 15,000	Ref.		Ref.	
≥15,000	1.780 (0.853–3.713)	0.124	0.775 (0.284–2.114)	0.619

Knowledge scores (1 = < 9 scores, 2 = ≥ 9 scores). *OR* Odds ratio, *CI* Confidence interval. Significant at *p*-value < 0.05

### Associations between attitudes and socio-demographic characteristics

As Table 4 shows the bivariate analysis found a significant association between attitudes and education at or beyond the bachelor’s degree as well as the age group of 31–35 years (*p*-value < 0.05). In addition, working as a regular employee and being in the 26–30 years age group were nearly associated with attitudes. The multivariate analysis found that individuals with at least a bachelor’s degree had more favorable attitudes about COVID-19 than those with less education (*p*-value = 0.040; OR = 2.733, 95%CI 1.045–7.149). In addition, those aged 26–30 and 31–35 years had more favorable attitudes than those aged 25 years or younger (OR = 2.518–2.664, 95%CI 0.841–8.442).

**Table 4:** Bivariate and multivariate analysis of attitudes and socio-demographic characteristics

Attitudes				
**Factor variables**	**Bivariate**		**Multivariate**	
	**OR (95%CI)**	***p*-value**	**OR (95%CI)**	***p*-value**
**Occupation**				
Civil servant	Ref.		Ref.	
Employee	0.370 (0.127–1.077)	0.068	0.646 (0.199–2.113)	0.478
Housewife	0.438 (0.140–1.370)	0.156	0.988 (0.254–3.840)	0.986
**Age (years)**				
18–25	Ref.		Ref.	
26–30	2.167 (0.917–5.110)	0.078	2.518 (0.946–6.702)	0.065
31–35	3.021 (1.184–7.708)	**0.021**	2.664 (0.841–8.442)	0.096
≥ 36	1.929 (0.743–5.009)	0.177	1.869 (0.592–5.900)	0.286
**Education (degree)**				
< Bachelor	Ref.		Ref	
≥ Bachelor	2.296 (1.129–4.670)	**0.022**	2.733 (1.045–7.149)	**0.040**
**Status**				
Unmarried	Ref.		Ref.	
Married	1.534 (0.738–3.187)	0.251	0.990 (0.382–22.569)	0.984
**Income (Thai baht)**				
< 15,000	Ref.		Ref	
≥15,000	1.296 (0.654–2.569)	0.457	0.628 (0.245–1.611)	0.333

Attitudes scores (1 = < 27 scores, 2 = ≥ 27 scores). *OR* Odds ratio, *CI* Confidence interval. Significant at *p*-value < 0.05

### Associations between practices and socio-demographic characteristics

As Table 5 shows, the bivariate analysis found that the age group of 26–30 years and income ≥15,001 were significantly associated with practices (*p*-value < 0.05). The multivariate analysis found that participants with at least a bachelor’s degree reported more proper practices than those with less education (*p*-value = 0.04; OR = 2.285 95%CI 1.110–6.146), and higher income was associated with higher practice scores (*p*-value = 0.005; OR = 4.747 95%CI 1.588–14.192). The age group of 26–30 years was more closely associated with higher practice scores than ages 25 years and below (OR = 2.413 95%CI 0.882–6.602).

**Table 5:** Bivariate and multivariate analysis of practices and socio-demographic characteristics

Practice				
**Factor variables**	**Bivariate**		**Multivariate**	
	**OR (95%CI)**	***p*-value**	**OR (95%CI)**	***p*-value**
**Occupation**				
Civil servant	Ref.		Ref.	
Employee	0.969 (0.349–2.693)	0.952	0.674 (0.200–2.275)	0.525
Housewife	0.690 (0.227–2.097)	0.513	0.649 (0.161–2.613)	0.542
**Age (years)**				
18–25	Ref.		Ref.	
26–30	2.629 (1.103–6.262)	**0.029**	2.413 (0.882–6.602)	0.086
31–35	1.197 (0.457–3.135)	0.714	1.167 (0.345–3.950)	0.804
≥ 36	2.133 (0.815–5.581)	0.123	2.314 (0.700–7.650)	0.169
**Education (degree)**				
< Bachelor	Ref.		Ref	
≥ Bachelor	2.256 (1.127–4.650)	**0.035**	0.285 (1.110–6.146)	**0.040**
**Status**				
Unmarried	Ref.		Ref.	
Married	1.141 (0.548–2.376)	0.725	0.717 (0.263–1.953)	0.515
**Income (Thai baht)**				
< 15,000	Ref.		Ref	
≥15,00	2.041 (1.020–4.082)	**0.044**	4.747 (1.588–14.192)	**0.005**

Practice scores (1 = < 18 scores, 2 = ≥ 18 scores). *OR* Odds ratio, *CI* Confidence interval. Significant at *p*-value < 0.05

### Correlation between knowledge, attitude, and practice scores

Table 6 indicates a weak but significant positive correlation between knowledge and practice scores (*r* = 0.210, *p*-value = 0.010). The correlation between attitude and practice scores approached significance (*r* = 0.159, *p*-value = 0.052).

**Table 6:** Spearman correlation analysis between knowledge, attitudes, and practices

Correlation coefficient (r)		
Variables	Practice	***p***-value
Knowledge	0.210	**0.010**
Attitudes	0.159	**0.052**
Practice	1	–

Correlation coefficient significant at *p*-value < 0.05

## Discussion

This study was conducted during the third wave of the COVID-19 pandemic in Thailand. To the best of our knowledge, it is the first study performed with pregnant women in an urban community. We found that socio-demographic factors such as occupation, age, education, marital status, and income influenced the knowledge, attitudes, and practices of pregnant women living in Bangkok. In addition, we found that knowledge score significantly increased along with practice scores, and attitude scores were nearly significantly correlated with practice scores. In addition, the best result reported that they had never been in a situation in which they risked contracting COVID-19 (84.7%). However, this study is based on the current situation of women pregnant during the COVID-19 pandemic in Bangkok Thailand.

### Association between knowledge and socio-demographic characteristics

Our study found most participants knew that COVID-19 can be spread by droplets and aerosols, this result is agreed with other reported studies [24]. Our finding over responded that the pneumococcal vaccine cannot protect against COVID-19, was likely reported from WHO that pneumococcal vaccine does not provide protection against the new coronavirus [25]. Knew that there is currently no treatment for COVID-19 and reported that regular rinsing nasal mucus with saline cannot prevent infection, previously result suggested that limited clinical evidence concerning the curative or preventive role of saline water gargling and nasal irrigation against COVID-19 infection [26]. However, participants responded that wearing two masks are more effective than a single mask to prevent infection, was likely agreed with previous study [27].

The multivariate analysis indicated that marital status was significantly associated with knowledge about COVID-19, whereby married participants exhibited a 4.9-fold increase of knowledge scores over unmarried participants. This finding is in agreement with previous studies [28, 29]. Although having a higher education degree was not significantly associated with knowledge, participants with at least a bachelor’s degree showed a 1.4-fold increase of knowledge scores over those with less education. It is important for clinicians to keep less educated pregnant women informed on preventative measures and provide them with psychological support [16]. In addition, Interventions are needed to improve educational levels of girls and women in the region [30].

### Association between attitudes and socio-demographic characteristics

Our study found most participants were aware that pregnancy could increase their risk of COVID-19 infection and intended to self-isolate for 2 weeks if they became infected after delivery, the WHO has concluded that mothers with suspected or confirmed COVID-19 should not be separated from their infants, but should rather practice respiratory hygiene and wear a mask, wash their hands before and after touching their infant, and routinely clean and disinfect surfaces [27, 31, 32]. This study found that most participants intended to breastfeed unless they became infected with COVID-19, which aligns with the results of a previous study [33, 34]. One study has emphasized the need to address pregnancy and breastfeeding in the ongoing pandemic [35, 36]. However, the WHO suggests that mothers and infants should be kept together regardless of COVID-19 status but that mothers should wash their breasts before every feeding in addition to the aforementioned measures [34]. Mothers who are too ill to breastfeed should explore alternatives infant such as using donated human milk or formula as a last resort [34]. In addition, previously suggested that knowledge about COVID-19 infection in pregnant women and newborns is scarce [18]. Moreover, our study found most participants that there is a very high percentage of pregnant women who would agree to be vaccinated of COVID-19 during pregnancy (52.7%), as other studies have reported low acceptability rates [21, 37, 38]. In addition, some study suggestion that many pregnant women are still reluctant to get vaccinated, vaccination is an effective and safe protective measure in these special population and should be encouraged by healthcare professionals and immunization programs should be organized by the states [39, 40].

The multivariate analysis found that participants with at least bachelor’s degree showed a 2.7-fold increase of favorable attitudes over less educated participants, and those aged 26–30 and 31–35 years showed a 2.5-fold increase in favorable attitudes over those aged 25 years and younger. One study has argued that providing relevant and reliable information along with comprehensive counseling is crucial for alleviating the psychological effects of the COVID-19 pandemic on pregnant women [33]. Hence, strategies should focus on reducing fear and improving attitudes toward the care of COVID-19 patients as well as the promotion of preventive practices [41, 42].

### Association between practices and socio-demographic characteristics

Our study found most participants reported wearing a mask every time they left the house and frequently washing their hands with soap and water or cleaning them with alcohol. Our findings are agreement with previous studies in which most participants expressed positive attitudes towards hand hygiene; however, a considerable gap was evident between attitudes and knowledge and reported hand hygiene behavior [43]. Most participants in this study reported avoiding crowds and public places as well as following news updates on the COVID-19 situation. Another study found that doctors, nurses/midwives, and the television were the most trusted sources of COVID-19 information among pregnant women [11].

The multivariate analysis found that a 2.2-fold increase in practice scores among participants with at least a bachelor’s degree compared with those with less education One study of pregnant women’s behaviors found that most participants paid close attention to news updates on COVID-19 [44]. In addition, there was a 4.7-fold increase in practice scores among participants reporting higher incomes. One study found that half of the respondents reported experiencing a significant decline in income during the pandemic [45]. Our study found a 2.4-fold increase of practice scores among participants aged 26–30 years compared with those aged 25 years and younger. A previous study suggested that healthcare providers need to counsel pregnant women aged 35 years and older on COVID-19 prevention practices [46].

### Correlation between knowledge, attitudes, and practices

We found a weak but significant positive correlation between knowledge and practice scores (*r* = 0.210, *p*-value = 0.010). This result aligns with previous research in which the majority of pregnant women reported healthy practices and adequate knowledge related to the COVID-19 pandemic [47–49], as well as studies showing that urban residents were more likely to evince adequate knowledge and practices [50, 51]. Special consideration should be given to people living in rural areas and less educated women. In addition, our study showed that attitude scores were not significantly correlated with practice scores, was recommend that interventions to improve and attitude and practice scores (*r* = 0.159, *p*-value = 0.052). Another study found gaps between COVID-19-related attitudes and practices among pregnant Syrian refugees and advocated for further health education measures [52]. Implications for practice include the need for health care providers to consider the impact of the pandemic on patients’ mental status, access to resources and behaviors [53, 54]. Future interventions and policies should emphasize a “person-centered” approach that targets and embraces vulnerable subgroups to close the gaps in COVID-19-related KAP and should be educated and advised about physiological and immunological changes in pregnancy make women more susceptible to severe illness from respiratory infections.

### Limitations

This study was a cross-sectional study conducted at a single hospital; it does not reflect the general population, which may introduce selection bias. The questionnaire responses were based on the perceptions and experiences of a specific group of current situations during the COVID-19 pandemic. Finally, we did not evaluate the questionnaire’s reliability because its aim was to measure participants’ perceptions of whether measures such as social distancing and other restrictive measures would enable the country to win the fight against the COVID-19 pandemic.

## Conclusion

During the third wave of the COVID-19 pandemic in Thailand, the pregnant women who participated in this study evinced overall high levels of knowledge, attitudes, and practices. Our study identified several influencing factors related to pregnant women’s COVID-19-related knowledge, attitudes and practices, and the results can guide public health strategies. In particular, we recommend that interventions to improve knowledge on pregnancy should focus on reducing fear and improving attitudes toward the care of COVID-19 patients as well as the promotion of preventive practices.

## Supplementary Information

**Additional file 1.** The full English language version of the questionnaire.

## Data Availability

The data sets generated and analyzed during the current study are not publicly available due to identifiable information but are available from the corresponding author on reasonable request answering the survey.

## Abbreviations

COVID-19: Coronavirus disease
KAP: Knowledge, Attitudes, Practices
ICU: Intensive Care Unit
WHO: World Health Organization
OR: Odds Ratio
CI: Confidence Interval
*r*: Correlation coefficient
