# Supplementary material for: Knowledge, attitudes, and practices related to the COVID-19 pandemic among pregnant women in Bangkok, Thailand
Source: BMC Pregnancy Childbirth. 2022 Apr 23;22:357. doi: 10.1186/s12884-022-04612-3 (PMC9034254; doi:10.1186/s12884-022-04612-3)
Supplement: Supplementary file 1 — Additional file 1. The full English language version of the questionnaire. [file 12884_2022_4612_MOESM1_ESM.docx]

**Additional file 1**

The full English language version of the questionnaire. The full English language version of the questionnaire contained all the details of the original Thai version of the questionnaire

**Questionnaire (English version)**

**Consent from**

**( ) Allow ( ) Not allow**

**ID ………………**

**The questionnaire is divided into four sections.**

**Section 1.**

Socio-demographic information, including age, occupation, education level, marital status, income, and religion, in addition to clinical characteristics such as pregnancy trimester, number of pregnancies, type of conception, history of miscarriages, current and previous pregnancy complications, settlement type, and questions designed to gauge participants’ risk of contracting COVID-19.

1. Age (years) ………………………………………….
2. Occupation

( ) Civil servant ( ) Employee ( ) Housewife

1. Education

( ) < Bachelor’s degree ( ) ≥ Bachelor’s degree

1. Status

( ) Unmarried ( ) Married

1. Income (Thai baht)

( ) ≤15,000 ( ) ≥15,001

1. Religion

( ) Buddhism ( ) Christianity ( ) Islam

1. Trimester

( ) First ≤12 weeks gestation

( ) Second 13–26 weeks gestation

( ) Third 27–40 weeks gestation

1. Number of pregnancies

( ) 1 ( ) 2 ( ) 3 ( ) ≥4

1. Type of conception

( ) Naturally conceived ( ) Not naturally conceived

1. Number of miscarriages

( ) 0 ( ) 1 ( ) ≥2

1. Current complications

( ) No ( ) Yes

1. Previous complications

( ) No ( ) Yes

1. Settlement type

( ) Urban ( ) Others ………………

1. Risk of contracting COVID-19

( ) Infection COVID-19

( ) At risk (ever had a screening test)

( ) Never at risk

( ) Not sure

**Section 2.**

The knowledge section consists of 13 items (K1–K13) scored as False = 0 and True = 1.

| Knowledge | | True | False |
| --- | --- | --- | --- |
| K1 | Human-to-Human transmission of COVID-19 |  |  |
| K2 | COVID-19 can be spread by droplets and aerosols. |  |  |
| K3 | COVID-19 symptoms include mild fever, tiredness, dry cough, and muscle pain. |  |  |
| K4 | Everyone has the same risk of infection from COVID-19. |  |  |
| K5 | There currently is no treatment for COVID-19. |  |  |
| K6 | The pneumococcal vaccine can protect against COVID-19. |  |  |
| K7 | Not everyone with COVID-19 will have severe symptoms. But people with underlying or chronic diseases are more likely to have severe symptoms. |  |  |
| K8 | Wearing two of masks can prevent infection from COVID-19 better than one layer. |  |  |
| K9 | Wash your hands frequently |  |  |
| K10 | Regular rinsing nasal mucus with saline can prevented with COVID-19. |  |  |
| K11 | A person who comes into contact with someone infected with COVID-19 should be isolated immediately in 14 days. |  |  |
| K12 | Vaccination against COVID-19 prevents severe symptoms. |  |  |
| K13 | There currently is no information on the efficacy and safety of vaccinations against COVID-19. |  |  |

**Section 3.**

The Attitudes section consisted of 17 items (A1–A17) assessing perceptions of whether the country could win the fight against the COVID-19 pandemic, for which responses were scored as Agree = 2, Not Sure = 1, and Disagree = 0.

| Attitudes | | Agree | Not Sure | Disagree |
| --- | --- | --- | --- | --- |
| A1 | COVID-19 can be controlled |  |  |  |
| A2 | COVID-19 has affected daily life. |  |  |  |
| A3 | Pregnant women may have a higher chance than other populations of being infected with COVID-19. |  |  |  |
| A4 | Pregnancy may increase the risk of respiratory failure than other population. |  |  |  |
| A5 | I am concerned about being infected with COVID-19 during pregnancy. |  |  |  |
| A6 | I am concerned about being infected with COVID-19 following pregnancy. |  |  |  |
| A7 | Do you think the fetus can be infected? |  |  |  |
| A8 | Do you think your baby can be infected after birth? |  |  |  |
| A9 | Do you think the baby can be infected during delivery? |  |  |  |
| A10 | Contracting COVID-19 during pregnancy may increase the risk of miscarriage |  |  |  |
| A11 | Do you agree if your doctor will be advised you for caesarean section over a vaginal delivery if you are diagnosed with COVID-19? |  |  |  |
| A12 | If you are diagnosed have COVID-19, how likely do you think is the risk of infection to the baby after delivery |  |  |  |
| A13 | If infected with COVID-19 after delivery, will you isolate yourself for 2 weeks? |  |  |  |
| A14 | Will you breastfeed by yourself? |  |  |  |
| A15 | If infected with COVID-19 after delivery, will you breastfeed? |  |  |  |
| A16 | Do you want to be vaccinated against COVID-19 during pregnancy? |  |  |  |
| A17 | Do you want to be vaccinated against COVID-19 during breastfeeding? |  |  |  |

**Section 4.**

The Practices section consisted of 11 questions (P1–P11) scored as Practiced = 2, Not sure = 1, and Not practiced = 0.

| Practice | | Practiced | Not sure | Not practiced |
| --- | --- | --- | --- | --- |
| P1 | Will/have been tested for COVID-19 during pregnancy |  |  |  |
| P2 | Will be tested if you experience COVID-19 symptoms during pregnancy |  |  |  |
| P3 | You wear a mask every time you leave the house. |  |  |  |
| P4 | You frequently wash your hands or clean them with alcohol. |  |  |  |
| P5 | You cover your mouth and nose with your elbow or a cloth or tissue when you cough or sneeze |  |  |  |
| P6 | You maintain at least 1 meter distance from others in public places. |  |  |  |
| P7 | You avoid crowds and public places. |  |  |  |
| P8 | You follow news updates about COVID-19 situation |  |  |  |
| P9 | You have been to a risky place in the last 5 days |  |  |  |
| P10 | You have left the house wearing a mask within the last 5 days |  |  |  |
| P11 | You have strictly complied with government announcements. |  |  |  |
